# Effect of shape deprivation on retinal thickness in myopic mice using an OCT method

**DOI:** 10.3389/fnins.2023.1156990

**Published:** 2023-04-06

**Authors:** Ming-Ming Duan, Hui Liu, Yu-Lin Zhong

**Affiliations:** ^1^Department of ophthalmology, the First People's Hospital of Jiujiang City, Jiujiang, Jiangxi, China; ^2^Department of ophthalmology, Jiangxi Provincial People’s Hospital, The First Affiliated Hospital of Nanchang Medical College, Nanchang, Jiangxi, China

**Keywords:** form-deprivation myopia, retina thickness, optical coherence tomography, myopia mice, myopia

## Abstract

**Purpose:**

The purpose of this study was to study in retina thickness changes in myopic mice using optical coherence tomography (OCT).

**Methods:**

There were 18 mice in the form-deprivation myopia (FDM) group，in which the left eye was not treated as a control;18 untreated mice served as a normal control group. The diopter of all mice was measured 21 days after birth (P21), before form deprivation. After 4 weeks of form deprivation (P49), the refraction, fundus, and retinal sublayer thickness of all mice were measured.

**Results:**

After 4 weeks of form deprivation, the refractive power of the right eye in the FDM group was significantly higher than that in the left eye (*p* < 0.05). There was no significant change in the refractive power of the left eye in the FDM group compared with the normal control group. The retina, nerve fiber layer (NFL), inner nuclear layer (INL), and outer nuclear layer (ONL) in the right eye of the FDM group were significantly thinner than those of both the FDM and control groups (*p* < 0.05). There was no significant change in photoreceptor (PR).

**Conclusion:**

Our study highlights that the myopic mice have decreased R thickness, which might reflect the potential pathological mechanism of myopia.

## 1. Introduction

Myopia is one of the most common diseases in ophthalmology. The disease can affect almost all of the population and has reached epidemic levels, especially in East and Southeast Asia (Jung et al., 2012; Saw et al., 2002; He et al., 2004). It has become a major public health problem that needs to be solved urgently. It is estimated that, by 2050, nearly half of the world’s people will be suffering from myopia (Holden et al., 2016). With the increase in the incidence of early-onset myopia (Myrowitz, 2012), more and more people will develop high myopia and pathological myopia. Pathological myopia leads to blindness, such as glaucoma, retinal detachment, and macular hole. A previous study suggests that the occurrence of myopia is caused by the interaction of genetic and environmental factors (Baird et al., 2020), and the environmental factors include the close work or study time, outdoor activities time and the excessive sugar intake. Some studies suggest the hyperopic defocusing causes axial length growth, which might lead to the decreased retina thickness. However, the pathogenesis is still unclear. At present, low concentrations of atropine and orthokeratology have achieved results in controlling the development of myopia (Bullimore & Richdale, 2020). However, the pathological structural changes in intraocular structure of the sclera, choroid, and retina (R) caused by myopia are irreversible (Lee et al., 2020; Jonas & Panda-Jonas, 2019).

Optical coherence tomography (OCT) is a non-invasive imaging method *in vivo*, which provides a high-resolution image of the retina, and can be used for quantitative and qualitative evaluation of different regions and layers of retina and optic nerve (Bhende et al., 2018). It can also be used to evaluate the relationship between refractive state and retinal thickness (Bonnin et al., 2015). OCT suggests that the retina and choroid will become thinner in myopic children (Matalia et al., 2018), and with the increase of axial length, the retinal nerve fiber layer (NFL) becomes thinner (Leung et al., 2006). As in myopia, atrophy around the optic papilla and thinning of the retinal NFL can be observed. However, there are also studies that suggest the opposite, in which the whole and temporal NFL of high myopia is thicker than that of the normal control group (Hsu et al., 2013). Therefore, our study of the changes of retinal structure and thickness might reveal the potential pathological mechanism of myopia, providing a new direction for the prevention and control of myopia.

The emergence of myopic animal models has promoted greatly the research on the occurrence, development, and treatment of myopia. Form deprivation in childhood can induce the change of myopia refractive state and prolongation of eye axis. This phenomenon has been confirmed in monkeys, sloths, marmosets, mice, guinea pigs, and chickens (Wiesel & Raviola, 1977; Schaeffel et al., 1988; Barathi et al., 2008; Shaikh et al., 1999; Graham & Judge, 1999), and it provides a basis for the establishment of a suitable animal model of myopia. Mice have become the first choice of myopic animal model because of the possibility of genetic manipulation, abundant available antibodies, and low-cost breeding. A large number of experiments have studied the changes of intraocular structure in myopic mice. However, there are few studies on the interlamellar thickness of the retina in myopic mice. The hypothesis of the study is that the form-deprivation myopia (FDM) causes the changes of retinal interlamellar thickness. Based on the hypothesis, our study is to establish a mouse model of FDM and use OCT method to study the changes of retinal interlamellar thickness in myopia.

## 2. Materials and methods

### 2.1. Animals

The study was approved by the Ethics Committee of Jiangxi Provincial People’s Hospital (Nanchang, China). The experimental procedure adhered strictly to the ARVO Statement for the Use of Animals in Ophthalmic and Vision Research.

In our study, 36 male C57BL/6 wild type mice (3 weeks old) were obtained from the Jiangxi Laboratory Animal Center. Mice were examined clinically to confirm no injuries or infections to the eyes and the refractive level remained consistent. After being divided randomly into 2 groups, all animals were kept at a constant temperature of 25 deg. Celsius. A 12-h light–dark cycle provided the light conditions for visual development in mice. Refractive errors in the FDM group (n = 18) were induced by wearing a translucent diffuser on the right eye from postnatal day 21 (P21). The contralateral untreated eyes in the FDM group served as a self-control group. The remaining untreated mice (n = 18) served as the control group.

### 2.2. Form deprivation

After anesthetizing with an injection of avertin, the model of shape deprivation myopia was constructed by covering the right eye of FDM group (n = 18) with a handmade translucent occluded device for 4 weeks. A collar made of thin plastic was attached to the neck to prevent the mice from removing the translucent balloon. For both the FDM group and control group, weight, refraction, and R were measured before and at the end of the 4-week treatment. After 4 weeks of form deprivation, mice with corneal ulcers and lesions were removed. To minimize measurement errors, all measurements were performed by one researcher.

### 2.3. Biometric measurements

Unanesthetized mice were placed in a dark room and their refractive status was measured with an infrared eccentric photorefractor at a working distance of 50 cm. At 3 min before the measurement, the mice were given eye drops of tropicamide in both eyes to dilate the pupils and facilitate the measurement of refractive status. After gently limiting the movement of the mouse, the head position of the mouse was adjusted to image the center of the pupil successfully, and the eyeball diopter number was read at this time. Each mouse was measured 5 times alternately in both eyes and averaged.

In the present study, we used OCT (Phoenix, MICRON IV) to measure the thickness of R in the mouse eye *in vivo*. After anesthetizing with an injection of avertin, the test eye was dilated with tropicamide, and ofloxacin ointment was applied to the corneal surface to facilitate clear fundus imaging. To improve image clarity, each OCT image is composed of 50 instantaneous layers. After OCT image acquisition, retinal stratification and thickness analysis were performed using Insight software. The thickness of different parts of the mouse R was different; here we focused on the 200 μm area adjacent to the optic nerve for analysis.

### 2.4. Statistical analysis

Data analysis was conducted using SPSS version 19.0. The deprived eyes and contralateral eyes of FDM mice were compared by paired t-test. Independent t-test was applied to compare the control and FDM eyes. After confirming the normal distribution of the data (Shapiro–Wilk test), analysis of variance (ANOVA) was used to compare the data of each parameter at different times or the four sets of data. Kruskal-Wallis test was used for data that did not conform to the normal distribution. In our study, data were expressed as mean ± standard deviation (SD) in the table or as mean ± SD in the figure. *P*<0.05 was considered statistically significant.

## 3. Results

**Figure 1:**
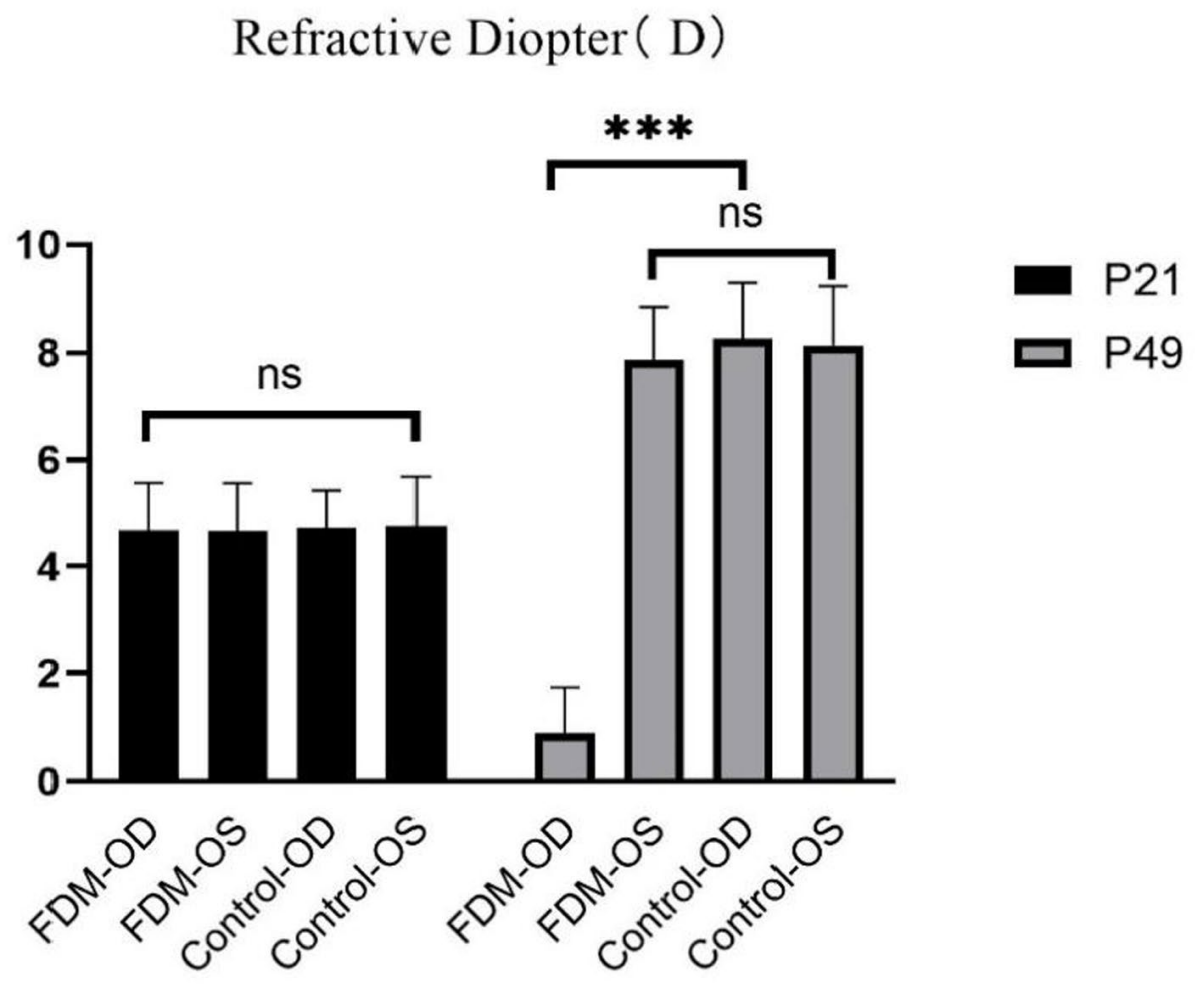
Refractions between the FDM and control groups. FDM: form-deprivation myopia; OD: oculus dexter; OS: oculus sinister.

**Figure 2:**
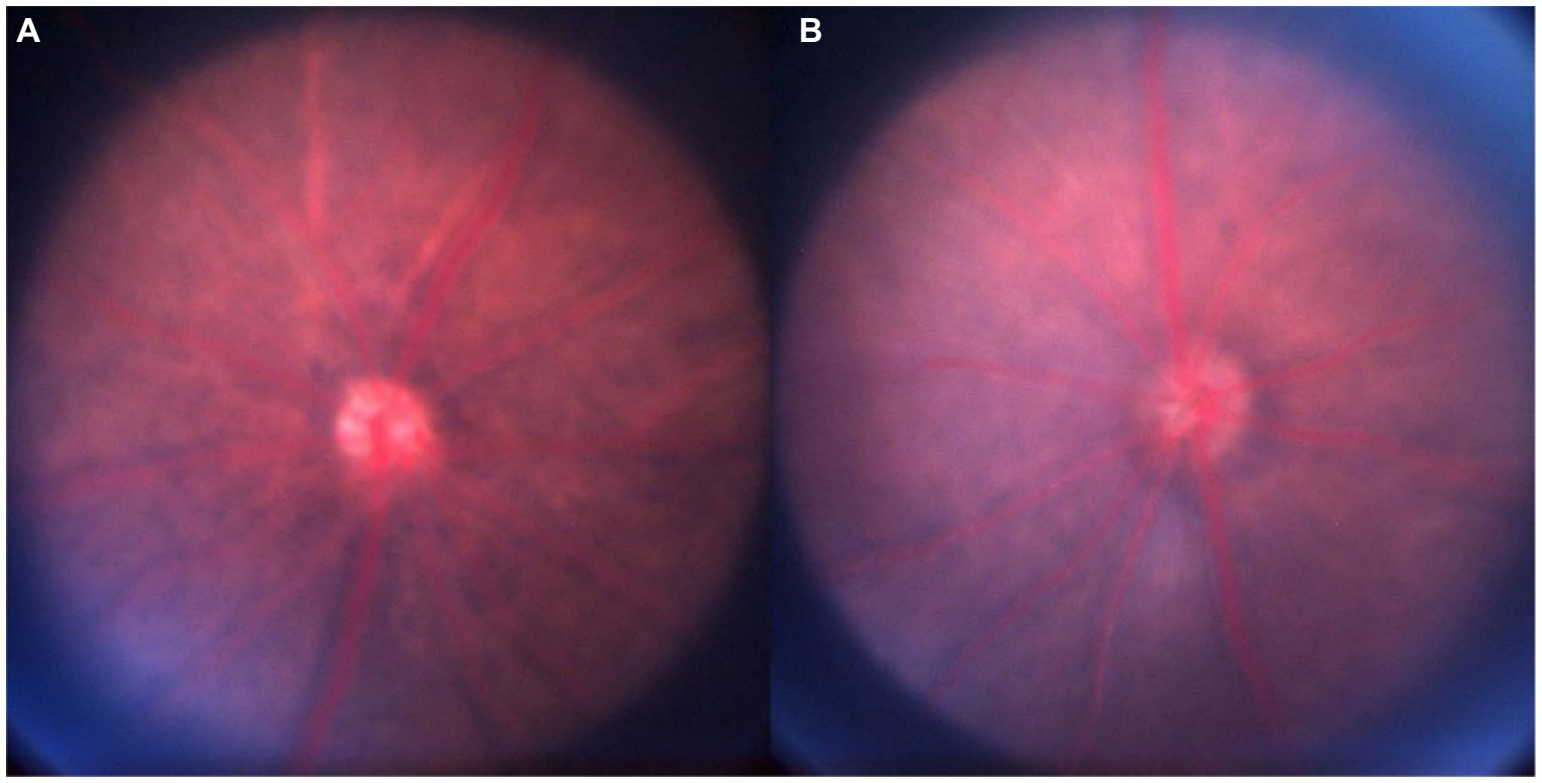
Fundus imaging. Notes: **(A)**: FDM-OD; **(B)**: FDM-OS. FDM: form-deprivation myopia; OD: oculus dexter; OS: oculus sinister.

**Figure 3:**
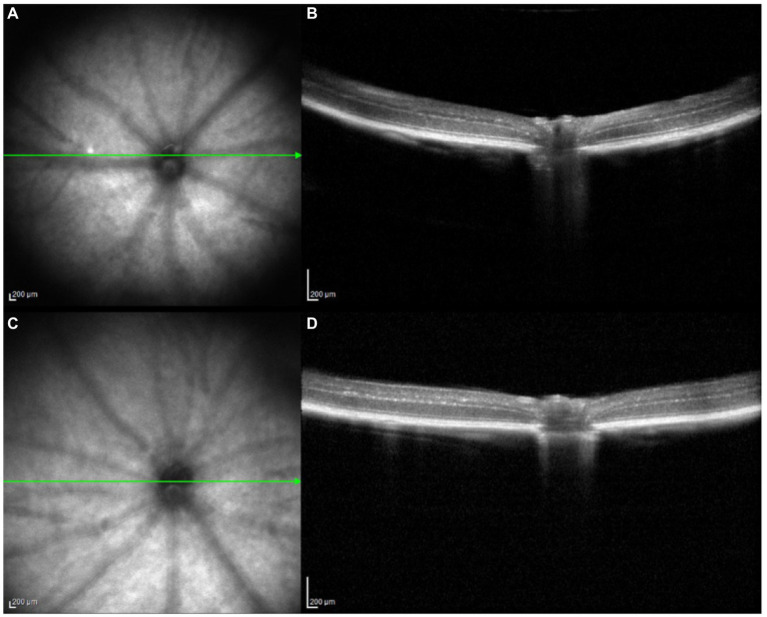
OCT images for the FDM-OD and FDM-OS. Notes: **(A, B)**: FDM-OD; **(C, D)**: FDM-OS. FDM: form-deprivation myopia; OD: oculus dexter; OS: oculus sinister; OCT: optical coherence tomography.

**Figure 4:**
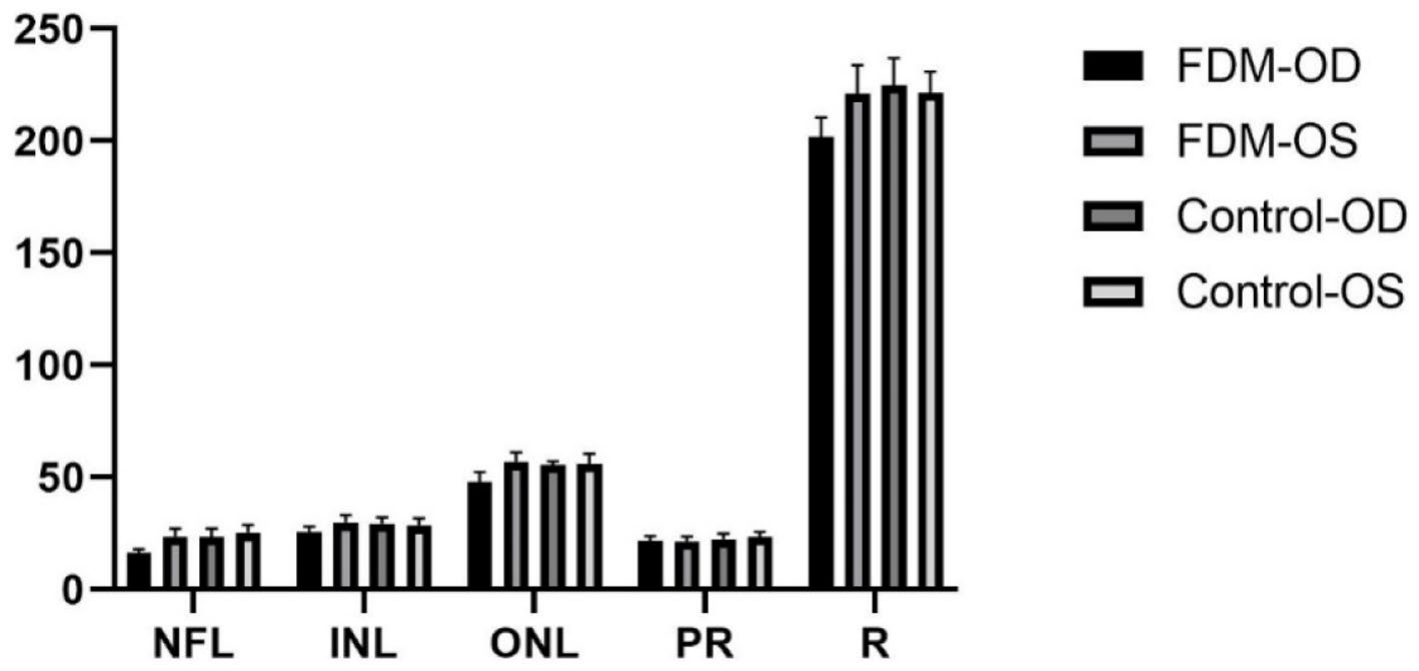
Thickness of the retina between the FDM and control groups. FDM: form-deprivation myopia; OD: oculus dexter; OS: oculus sinister; R: retina; NFL: nerve fiber layer; INL: inner nuclear layer; ONL: outer nuclear layer; PR: photoreceptor.

As shown in Figure 1 and Table 1, after 4 weeks of continuous form deprivation treatment in FDM group, the refractive power between the left and right eyes was significantly different in the FDM group. There were no statistically significant differences among left eyes in the FDM group and bilateral eyes in the control group. Figure 2 showed the fundus of left and right eyes of mice in FDM group after 4 weeks of form deprivation treatment, without obvious abnormal indications. Figure 3 showed the OCT images of the right and left eyes of mice in the FDM group after 4 weeks of treatment. Combined with Table 2, it could be seen that the retinal thickness (include NFL, INL, and ONL) of the right eye of mice in the FDM group was significantly thinner than that of the left eye and the control group (*P*<0.001). Figure 4 showed the retinal thickness of each layer in the bar graph between two groups.

**Table 1:** Refractions between the FDM and control groups.

group	n	Diopter (D)	
		P21	P49
FDM-od	18	4.68 ± 0.89	0.88 ± 0.84
FDM-os	18	4.66 ± 0.90	7.87 ± 0.96*
Control-od	18	4.72 ± 0.70	8.26 ± 1.02*
Control-os	18	4.76 ± 0.93	8.14 ± 1.08*
F		0.048	241.670
*P*		0.986	0.000

FDM: form-deprivation myopia; OD: oculus dexter; OS: oculus sinister. * indicate P < 0.05

**Table 2:** Thickness of the retina between the FDM and control groups (mean ± s，μm).

Groups	n	R	NFL	INL	ONL	PR
FDM-od	18	201.97 ± 8.59	16.36 ± 1.76	25.45 ± 2.55	47.99 ± 4.49	21.70 ± 2.19
FDM-os	18	220.93 ± 12.83*	23.58 ± 3.61*	29.98 ± 3.17*	57.08 ± 4.09*	21.48 ± 2.17
Control-od	18	224.65 ± 12.38*	23.39 ± 3.75*	29.53 ± 2.73*	55.46 ± 1.68*	22.33 ± 2.50
Control-os	18	221.44 ± 9.56*	25.28 ± 3.67*	28.62 ± 3.20*	56.04 ± 4.53*	23.42 ± 2.29
F		15.862	38.376	22.314	26.967	2.574
*P*		0.000	0.000	0.000	0.000	0.061

FDM: form-deprivation myopia; R: retina; NFL: nerve fiber layer; INL: inner nuclear layer; ONL: outer nuclear layer; PR: photoreceptor; OD: oculus dexter; OS: oculus sinister.

* indicate p < 0.05.

## 4. Discussion

At present, researchers have successfully established common myopia models such as monkey, tree shrew, mouse, guinea pig, and chicken. There are differences in the structure and developmental characteristics of the eyeball in these common myopia models, so their applications in myopia research are also different. Mice are easy to obtain and raise, and their eyeball structure is similar to that of humans. In addition, the mouse is highly fertile and has complete genome information and mature genetic manipulation methods, which make this animal model widely used in myopia research. There are two main types of classical myopia induction methods: form-deprivation myopia (FDM) and lens-induced myopia (LIM). In this study, we found the R, NFL, inner nuclear layer (INL), and outer nuclear layer (ONL) around the optic nerve were thinner in myopic mice with FDM method. Thinning of the R caused by myopia has been reported in many studies, with a large number of studies suggesting that the R thins in myopia (Xie et al., 2009; Zereid & Osuagwu, 2020). However, some studies have reported an increase in retinal thickness in the central macular area of high myopia (Guo et al., 2020; Kim et al., 2019). Further analysis of the thickness of different retinal layers showed that the macular ganglion cell complex (GCC)(Guo et al., 2019), NFL (Ucak et al., 2020), and inner plexiform layer (Lee et al., 2020) were also thinned in myopia. Because previous studies on myopic retinal thickness changes are controversial. To clarify this issue, in this study, we used OCT to analyze the interlayer thickness of the eyes in myopic mice because it is non-invasive and can obtain high-definition images of the intraocular cross-section. Form deprivation caused the thinning of all retinal layers. The number of cells in the nucleated cell layer, photoreceptor cell layer, inner nuclear cell layer and ganglion cell layer decreased, and their arrangement was sparse and disordered. The thinning of the R might be caused by the stretching effect of the increase of the eye axis or by the thinning of different layers of the R.

The thickness of the NFL around the optic nerve in this study was significantly lower than that in the control group. Similar findings have been found in other myopic animals, such as thinning of the NFL in myopic chickens (Swiatczak et al., 2019), and patients with high myopia also have progressive loss of R NFL around the optic nerve papilla (Chopra & Lee, 2019), with some studies suggesting that the thinning of NFL is related to the blood perfusion of the R and the decrease of microvessels (Wang et al., 2016; Ucak et al., 2020; Li et al., 2017), and it is suggested that there is a correlation between retinal ischemia and NFL thinning. Myopia is a risk factor for open-angle glaucoma and studies have shown that the damage of the NFL structure often occurs before the visual-field damage and the thinning of the NFL in myopia might also affect the development of glaucoma in some young patients (Lee et al., 2017). The length of eye axis is correlated negatively with the thickness of NFL and the thinning of the NFL might be a result of the stretching effect of eye axis growth (Ganekal et al., 2021). However, contrary to our results, an earlier study suggests that only the nasal NFL is lower than normal, the thickness of the NFL of the upper and lower nasal side is normal, and even the whole and temporal NFL is thicker than normal (Hsu et al., 2013).

Our study found that the INL and ONL around the optic nerve were also significantly thinner. Similar changes were found in other animal models of form deprivation myopia. For example, INL thinning was found in myopic tree shrew and chickens (Mao et al., 2006; Abbott et al., 2011). Similar to our results, the thickness of the inner and outer layers of the R decreased in high myopia and thinning of the INL and outer plexiform layer (OPL) was also found in anisometropia myopia (Kim et al., 2020; Kirik et al., 2021). Some studies have suggested that the thinning of the inner R might be caused by the tangential tensile force caused by the elongation of the axial length，and the thinning of the outer R is caused by anterior and posterior traction (Wu et al., 2008). Recent studies have found that increased release of dopamine in myopic chicks leads to cell activation and cell density of bipolar cells and amacrine cells in INL (Mathis et al., 2020). The thinning of the INL might also be the atrophy caused by the decrease of the activation of retinal inner layer cells in myopia.

The ONL is composed mainly of cell bodies of rods and cones and our study suggests that ONL becomes thinner. Similar to our results, studies have confirmed that ONL is thinner in high myopia and the packing density and regularity of cones in the outer layer of the R decreased (Park et al., 2013; Wang et al., 2019). As the axial length lengthens, the elongation and thinning of the R might lead to sparse cones and the choroid of myopia will become thinner and the blood flow will decrease (Zhang et al., 2019; Wong et al., 2017). The oxygen supply of the outer layer of the R is mostly provided by the choroid, therefore, the thinning of the outer layer of the R might also be caused by hypoxia caused by myopia. Some studies have suggested that the change of the thickness of the outer retinal sublayer is related to the microvessel density in the R (Ye et al., 2020). Our study suggests that the changes of photoreceptor are not statistically significant, but, unlike our results, there are other studies that suggest that the photosensitive layer of myopia becomes thinner (Chen et al., 2012) and, in pathological myopia, the myoid and ellipsoid zone also becomes thinner (Ye et al., 2020).

The study still has some shortcomings in the study. First, anterior segment OCT scanning should be performed on myopic mice. This measurement captures a number of indicators of the overall structure of the eye, including axial length, central corneal thickness, anterior chamber depth, retinal thickness, and corneal curvature radius. Secondly, the sample size should be expanded and the success rate of FDM modeling should be improved. Finally, in future experiments, we should not only test the mouse model, but also the guinea pig or macaque model which is more similar to the human visual system, so as to increase the universality and conviction of the theory.

In conclusion, our study highlights the decreased retina thickness in myopia mice, which might reflect the potential pathological mechanism of myopia.

## Data availability statement

The raw data supporting the conclusions of this article will be made available by the authors, without undue reservation.

## Ethics statement

The animal study was reviewed and approved by the medical ethics committee of the Jiangxi Provincial People’s Hospital.

## Author contributions

M-MD, HL, and Y-LZ contributed to data collection, statistical analyses, wrote the manuscript, designed the protocol, contributed to the MRI analysis, designed the study, oversaw all clinical aspects of study conduct, and manuscript preparation. All authors contributed to the article and approved the submitted version.

## Conflict of interest

The authors declare that the research was conducted in the absence of any commercial or financial relationships that could be construed as a potential conflict of interest.

## Publisher’s note

All claims expressed in this article are solely those of the authors and do not necessarily represent those of their affiliated organizations, or those of the publisher, the editors and the reviewers. Any product that may be evaluated in this article, or claim that may be made by its manufacturer, is not guaranteed or endorsed by the publisher.
